# Insomnia, A Comorbidity to Take Into Account in the Global Management of Patients With Irritable Bowel Syndrome?

**DOI:** 10.1111/nmo.70336

**Published:** 2026-05-05

**Authors:** Eline Albert, Hiba Mikhael‐Moussa, Fabien Wuestenberghs, Anne‐Marie Leroi, Marie Netchitailo, Guillaume Gourcerol, Inês A. Trindade, Charlotte Desprez, Chloé Melchior

**Affiliations:** ^1^ Department of Gastroenterology, CIC‐CRB 1404 CHU Rouen Rouen France; ^2^ Nantes University Nantes France; ^3^ Univ Rouen Normandie INSERM, Normandie Univ, ADEN UMR1073, Nutrition, Inflammation and Microbiota‐Gut‐Brain Axis Rouen France; ^4^ Department of Gastroenterology and Digestive Oncology Sorbonne Paris Nord University, Hospital Avicenne, AP‐HP Bobigny France; ^5^ Department of Physiology, CIC‐CRB 1404 CHU Rouen Rouen France; ^6^ EMBRACE Lab, Center for Health and Medical Psychology (CHAMP), School of Behavioural Social and Legal Sciences University of Örebro Örebro Sweden; ^7^ University of Coimbra CINEICC ‐ Center for Research in Neuropsychology and Cognitive Behavioral Intervention, Faculty of Psychology and Education Sciences Coimbra Portugal

**Keywords:** insomnia, irritable bowel syndrome, prevalence, symptom severity

## Abstract

**Introduction:**

In the general population, many people are affected by both insomnia and irritable bowel syndrome (IBS). In this study, we aimed to explore the prevalence of insomnia in patients with IBS and its association with the severity of gastrointestinal and extra‐intestinal symptoms.

**Methods:**

Patients with IBS according to Rome IV criteria, referred to the Physiology Unit of Rouen University Hospital (France) between September 2022 and February 2025, who completed various validated questionnaires during their visit to assess insomnia (Insomnia Severity Index (ISI)), IBS severity (IBS‐SSS), anxiety and depressive symptoms (HAD‐S), quality of life (IBS‐QOL), upper gastrointestinal symptom severity (PAGI‐SYM), and somatic symptom severity (PHQ‐15), were prospectively included in our study. Patients were considered insomniacs when their ISI score was ≥ 15.

**Results:**

Among 700 patients with IBS included in our analysis, 270 (38.6%) were insomniac. The presence of insomnia was associated with more severe gastrointestinal symptoms (IBS severity, IBS‐SSS, *p* < 0.001) and somatic symptom severity (PHQ‐15, *p* < 0.001). The hierarchical linear regression analysis showed that the ISI score was associated with higher scores of anxiety and depressive symptoms, the presence of functional dyspepsia and fibromyalgia, and, to a lesser extent, older age and higher IBS severity scores (Adjusted R^2^ = 0.265).

**Discussion:**

Our findings suggest that in patients with IBS, insomnia is linked with psychological and somatic symptom burden, and the presence of comorbidities such as functional dyspepsia, but causality cannot be inferred. Future studies using objective sleep assessments, such as polysomnography, are needed to confirm these associations.

## Summary

- Anxiety and depressive symptoms, rather than IBS symptom severity, are associated with the presence of insomnia in patients with IBS.
- Patients with IBS and insomnia experience more severe gastrointestinal and extra‐intestinal symptoms and have more comorbidities.
- Insomnia appears to be associated with all IBS subtypes similarly.

## Graphical abstract

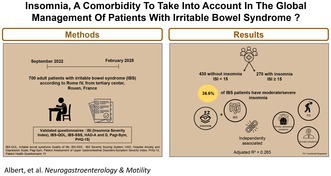

## 1 Introduction

According to Rome IV criteria, irritable bowel syndrome (IBS) is estimated to affect around 4% of the world's population [1] and is characterized by recurrent abdominal pain associated with transit disorders (diarrhea, constipation, or alternation of both). IBS symptoms are aggravated during painful outbreaks and can be exacerbated by the presence of comorbidities such as anxiety and depression [2]. Although this condition is benign from a medical point of view, it can severely impair patients' quality of life due to the discomfort and inconvenience it causes daily.

On the other hand, insomnia is estimated to affect 10% of the adult general population [3], but estimating its prevalence is difficult due to the heterogeneity of definitions and methods used [4]. This condition is characterized by difficulties in initiating or maintaining sleep, associated with early awakening, which has an impact on the following day. Indeed, these difficulties are usually accompanied by significant distress or alterations in daytime functioning, such as fatigue, concentration problems, or even irritability during the day [5]. Additionally, lack of restful sleep is known to increase pain sensitivity [6, 7].

Interestingly, 37.6% of patients with IBS are also affected by sleep disorders [8], which seems higher than in the general population.

These two conditions share overlapping pathophysiological pathways involving dysregulation of the gut‐brain‐microbiota axis, heightened stress reactivity, and impaired emotional regulation. An altered gut microbiota composition influences neuroendocrine signaling via the hypothalamic–pituitary–adrenal (HPA) axis, promoting systemic inflammation and heightened arousal. This state of hyperarousal, characterized by persistent sympathetic activation and disrupted circadian rhythms, exacerbates both gastrointestinal and sleep disorders [9, 10]. Thus, poor emotional regulation amplifies stress reactions and visceral sensitivity, creating a self‐perpetuating cycle linking gut dysfunction, sleep disturbances, and emotional distress.

Given the strong bidirectional communication between the gut, brain, and sleep systems, insomnia in IBS likely reflects dysregulation across physiological and psychological domains. Therefore, our study aimed to explore the prevalence of insomnia in patients with IBS and the factors associated with this overlap. We hypothesized that patients with IBS overlapping with insomnia have more severe gastrointestinal and extra‐intestinal symptoms and more comorbidities.

## 2 Materials and Methods

### 2.1 Patients

We included consecutive patients who were referred to our outpatient clinic (Digestive Physiology Unit, Rouen University Hospital, France) between September 2022 and February 2025 and fulfilled the Rome IV criteria for IBS. To be included, all participants had to be 18 years of age or older, have given a written informed consent, and have completed different validated self‐report questionnaires. All patients are part of the cohort from the research protocol OTFI “OBSERVATOIRE DES PATIENTS SOUFFRANT DE TROUBLES FONCTIONNELS INTESTINAUX”, approved by Ile‐de‐France VII Committee for the Protection of Persons board in 2019 (N° ID‐RCB: 2017‐A02134‐49). Our study was approved by the qualification committee of the Clinical Research and Innovation Department (DRCI) of the Rouen University Hospital under the reference number: 2025/0140 OB.

### 2.2 Measures

Relevant demographic information was collected: age, sex, body mass index (BMI), smoking, alcohol consumption, and recreational drug use. Validated questionnaires were used for patient data collection.

In order to divide our population into two groups of patients: with and without insomnia, the Insomnia Severity Index (ISI) was used. The ISI is a validated and widely used instrument for assessing insomnia severity in both clinical and research settings. It is a seven‐item questionnaire that assesses the severity of the patient's insomnia (difficulty falling asleep, staying asleep, and problem waking up too early), their satisfaction with their current sleep pattern, the interference of their sleep problems with daily functioning, the extent to which the impact of their sleep problem is noticeable to others, and the level of worry or distress they cause [11]. Each item ranges from 0 (e.g., no severity/no interference) to 4 (e.g., very severe, very dissatisfied). The total score ranges from 0 to 28. Scores of 8–14 indicate subthreshold insomnia, 15–21 indicate moderate insomnia, and 22–28 indicate severe insomnia. To identify patients with moderate to severe insomnia, a cutoff score of ≥ 15 was used; patients meeting this cutoff were classified as “IBS patients with insomnia”. This approach reduces the risk of misclassification associated with subthreshold symptoms and allows for the identification of patients with more clinically meaningful sleep disturbances.

Rome IV criteria were used for detecting disorders of gut‐brain interaction (DGBI) and in particular an overlap between IBS and FD [12].

The Irritable Bowel Syndrome Quality of Life Questionnaire (IBS‐QOL), consisting of 34 items, rating each question from 1 (very much) to 5 (not at all), was used for assessing quality of life. The questionnaire covered eight subscales: dysphoria, interference with activity, body image, health worry, food avoidance, social reaction, sexual, and relationship. Better quality of life is indicated by higher global scores, which range from 0 to 100 [13].

The IBS Severity Scoring System (IBS‐SSS), consisting of five items, each with a score ranging from 0 to 100, was used for assessing the severity and frequency of abdominal pain, severity of bloating, dissatisfaction with bowel habits, and daily life interference. The global score ranges from 0 to 500, with mild IBS severity ranging from 75 to 174, moderate from 175 to 299, and severe from 300 to 500 [14].

The Hospital Anxiety and Depression Scale (HAD‐S) was used for the assessment of anxiety (HAD‐A) and depressive symptoms (HAD‐D), with a total of 14 items (7 for each subscale). Each score ranges from 0 to 21. Scores from 0 to 7 indicate an absence of symptoms, 8 to 10 indicate a questionable presence of symptoms, and ≥ 11 indicate a definite presence of symptoms [15]. HAD‐A and HAD‐D scores were converted into qualitative variables (Yes/No) using a cut‐off score of 11. Scores < 11 indicated the absence of anxiety and/or depressive symptoms, whereas scores ≥ 11 indicated their presence.

The Patient Health Questionnaire‐15 (PHQ‐15) was used for the assessment of somatic symptom severity. It contains 15 items, each item ranging from 0 to 2, with a total PHQ‐15 score ranging from 0 to 30, and scores of 5, 10, and 15 indicating low, medium, and high somatic symptom severity, respectively [16].

The Patient Assessment of Upper Gastrointestinal Disorders‐Symptom Severity Index (Pagi‐Sym), consisting of 20 questions graded from 0 (no symptoms) to 5 (very severe symptoms), was used to assess the frequency and severity of symptoms associated with upper gastrointestinal disorders such as functional dyspepsia (FD), gastroesophageal reflux disease (GERD), and gastroparesis [17].

The presence of GERD and self‐reported extra‐intestinal comorbidities such as endometriosis and fibromyalgia were assessed by keywords from patients' medical records and surveys.

### 2.3 Statistical Analysis

Demographic and patient characteristics were first presented for the global study population. We then divided patients into two groups based on the presence or absence of insomnia and compared their characteristics.

Continuous variables are expressed as mean values ± standard deviation (SD), and categorical variables are expressed as number of patients and percentage. Chi‐square tests were used to analyze qualitative variables, and independent sample *t*‐tests were used for quantitative variables. A *p* < 0.05 was considered statistically significant. Cohen's d was used for measuring the effect size, quantifying the difference between two means. The closer the coefficient is to one, the greater the effect. We compared all data between the two groups: demographics, questionnaire scores, and comorbidities. Given the number of comparisons performed, these analyses were considered exploratory. No formal correction for multiple testing was applied, and results should therefore be interpreted with caution.

To better understand the determinants of insomnia severity, we performed a hierarchical linear regression using all factors significantly correlated with the ISI score (*p* < 0.05) as covariates, and the ISI as the dependent variable. The IBS‐QOL was not included, as it is a consequence of symptoms. By adding the independent variables in stages, we formed four models, which allowed us to better understand how much additional variance in the ISI score was explained by the new predictors in each model, and to control for broad and confounding variables. Model 1 included the broadest variables, related to demographic data. In Model 2, GI disorders were added, and IBS‐SSS was added in Model 3. The final Model 4 included extra‐intestinal symptoms. In the presented results of the hierarchical linear regression, B is the unstandardized predictor coefficient in the model, indicating the crude effect of an independent variable on the dependent variable. R^2^ is the total variance explained by the current model; Adjusted R^2^ corrects for the number of predictors in the model and the sample size, and F Change is the F test of variance gain.

For comparisons, correlations, and the hierarchical linear regression, we used IBM SPSS Statistics version 25. Excel 2016 was used for calculating Cohen's d.

## 3 Results

### 3.1 Global Study Population

We included 700 patients with IBS (mean age = 43.9 ± 15 and female gender = 78.6%). The majority of patients had an IBS overlapping with FD (60.9%) and IBS‐D was the predominant subtype in this cohort (33.3%). Our population had moderate IBS and somatic symptom severity (mean IBS‐SSS score: 254.8 ± 103.6, mean PHQ‐15: 13.4 ± 5.2), and is more affected by anxiety symptoms (36.9%) than depressive symptoms (18.7%) (Table 1). In our sample, 36.4% of patients had subthreshold insomnia (8 ≤ ISI ≤ 14), 30.4% had moderate insomnia (15 ≤ ISI ≤ 21), and 8.1% had severe insomnia (22 ≤ ISI ≤ 28).

**TABLE 1:** Patient characteristics, total population.

Characteristics	Values
*Demographics (n = 700)*	
Female	550 (78.6)
Age (year)	43.9 ± 15
BMI (Kg/m^2^)	25.3 ± 5.9
Smoking	136 (19.4)
Number of cigarettes per day	1.6 ± 4.5
Alcohol consumption	296 (42.3)
Number of glasses per week	1.4 ± 3.0
Recreational drug use	31 (4.4)
*Gastrointestinal comorbidities*	
IBS	700 (100.0)
IBS‐C	184 (26.3)
IBS‐D	233 (33.3)
IBS‐M	172 (24.5)
IBS‐U	111 (15.9)
FD	426 (60.9)
EPS only	116 (16.6)
PDS only	102 (14.6)
EPS + PDS	208 (29.7)
GERD	175 (25.0)
*Extra‐intestinal comorbidities*	
Anxiety symptoms	258 (36.9)
Depressive symptoms	131 (18.7)
Endometriosis	106 (15.1)
Fibromyalgia	87 (12.4)
*Questionnaires scores*	
IBS severity scoring system (IBS‐SSS)	254.8 ± 103.6
IBS quality of life (IBS‐QOL)	56.9 ± 23.4
Upper gastrointestinal disorders symptom severity (Pagi‐Sym, *n* = 660)	46.6 ± 17.9
Somatic symptom severity (PHQ‐15)	13.4 ± 5.2
Insomnia severity index (ISI)	12.4 ± 6.3

*Note:* Results are presented as mean ± standard deviation, or number (percentage). The number of cigarettes and alcoholic drinks consumed was calculated only for people who smoke and/or consume alcohol. Anxiety and Depressive symptoms are considered present if HAD‐A or HAD‐D score ≥ 11, respectively.

Abbreviations: BMI, Body Mass Index; EPS, Epigastric Pain Syndrome; FD, Functional dyspepsia; GERD, gastroesophageal reflux disease; IBS, Irritable Bowel Syndrome; IBS‐C, IBS with predominant constipation; IBS‐D, IBS with predominant diarrhea; IBS‐M, IBS with mixed bowel habits; IBS‐QOL, IBS Quality of Life; IBS‐SSS, IBS Severity Scoring System; IBS‐U, IBS unclassified; ISI, Insomnia Severity Index; Pagi‐Sym, Patient Assessment of Upper Gastrointestinal Disorders‐Symptom Severity Index; PDS, Postprandial Distress Syndrome; PHQ‐15, Patient Health Questionnaire‐15.

### 3.2 Characteristics of Patients With IBS and Insomnia

The prevalence of insomnia (moderate to severe) was 38.6% in our IBS population, which corresponds to 270/700 patients with an ISI score ≥ 15.

As described in Table 2, demographic data were similar between the two groups, but patients with insomnia were more likely to be smokers (*p* = 0.004). The distribution of IBS subtypes was equivalent between both groups (*p* = 0.58). Gastrointestinal (FD, *p* < 0.001 and GERD, *p* = 0.015) and extra‐intestinal (anxiety and depressive symptoms, *p* < 0.001, endometriosis, *p* = 0.028, and fibromyalgia, *p* = 0.014) comorbidities were more frequent in patients with insomnia. The insomnia group had greater symptom severity for gastrointestinal (IBS‐SSS, PAGI‐SYM) and somatic (PHQ‐15) symptoms (*p* < 0.001), and a lower quality of life (IBS‐QOL) (*p* < 0.001) compared to patients without insomnia. The most pronounced difference was observed for the severity of somatic symptoms (PHQ‐15, Cohen's d = 0.97).

**TABLE 2:** Comparison of IBS patients with and without insomnia.

Variable	IBS patients without insomnia	IBS patients with insomnia	*p*	Cohen's d
*Demographics*	*n* = 430	*n* = 270		
Female	337 (78.4)	213 (78.9)	0.87	
Age (year)	43.5 ± 15.2	44.7 ± 14.6	0.28	0.08
BMI (Kg/m^2^)	25.2 ± 5.8	25.4 ± 6.0	0.65	0.04
Smoking	69 (16.1)	67 (24.8)	**0.004**	
Alcohol consumption	193 (44.9)	103 (38.2)	0.08	
Recreational drug use	17 (4.0)	14 (5.2)	0.44	
	*n* = 69	*n* = 67		
Number of cigarettes per day	7.6 ± 7.0	9.3 ± 6.8	0.15	0.25
	*n* = 193	*n* = 103		
Number of glasses per week	3.1 ± 3.1	3.8 ± 5.1	0.18	0.19
*Gastrointestinal comorbidities*	*n* = 430	*n* = 270		
IBS‐C	115 (26.7)	69 (25.6)	0.58	
IBS‐D	98 (22.8)	74 (27.4)		
IBS‐M	146 (34.0)	87 (32.2)		
IBS‐U	71 (16.5)	40 (14.8)		
FD	232 (54.0)	194 (71.9)	**< 0.001**	
EPS	69 (16.0)	47 (17.4)	**< 0.001**	
PDS	64 (14.9)	38 (14.1)		
EPS + PDS	99 (23.0)	109 (40.4)		
GERD	94 (21.9)	81 (30.0)	**0.015**	
*Extra‐intestinal comorbidities*	*n* = 430	*n* = 270		
Anxiety symptoms	111 (25.8)	147 (54.4)	**< 0.001**	
Depressive symptoms	55 (12.8)	76 (28.1)	**< 0.001**	
Endometriosis	55 (16.3)	51 (23.9)	**0.028**	
Fibromyalgia	43 (10.0)	44 (16.3)	**0.014**	
*Questionnaire Scores*	*n* = 430	*n* = 270		
IBS severity scoring system (IBS‐SSS)	238.8 ± 104.7	280.2 ± 96.5	**< 0.001**	0.41
IBS quality of life (IBS‐QOL)	61.6 ± 22.3	49.5 ± 23.2	**< 0.001**	0.53
Somatic symptom severity (PHQ‐15)	11.7 ± 4.6	16.3 ± 4.9	**< 0.001**	**0.97**
	*n* = 404	*n* = 256		
Upper gastrointestinal disorders‐symptom severity (Pagi‐Sym)	43.3 ± 17.0	53.4 ± 17.1	**< 0.001**	0.59

*Note:* Results are presented as mean ± standard deviation, or number (percentage), *p* and Cohen's d bold values = significant.

Abbreviations: BMI, Body Mass Index (the number of cigarettes and alcoholic drinks consumed was calculated only for people who smoke and/or consume alcohol); Cohen's D, effect size; EPS, Epigastric pain syndrome; FD, Functional Dyspepsia; GERD, gastroesophageal reflux disease (Anxiety and Depressive symptoms are considered present if HAD‐A or HAD‐D score is ≥ 11, respectively); IBS, Irritable Bowel Syndrome; IBS‐C, IBS with predominant constipation; IBS‐D, IBS with predominant diarrhea; IBS‐M, IBS with mixed bowel habits; IBS‐QOL, IBS Quality of Life; IBS‐SSS, IBS Severity Scoring System; IBS‐U, IBS unclassified; PAGI‐SYM: Patient Assessment of Upper Gastrointestinal Disorders‐Symptom Severity Index; PDS, Postprandial distress syndrome; PHQ‐15, Patient Health Questionnaire‐15.

### 3.3 Predictors of Insomnia Severity in Patients With IBS

Next, we examined which variables were correlated with the ISI score in our patients (Table 3), to identify the variables to include in our linear regression. Eleven variables were significantly correlated with the ISI score (age, number of cigarettes per day, FD, GERD, fibromyalgia, endometriosis, IBS‐SSS, PHQ‐15, Pagi‐Sym, and HAD‐A, and HAD‐D), of which, three variables (PHQ‐15, HAD‐A, and HAD‐D) were moderately correlated with the ISI score (0.4 < *r* < 0.59).

**TABLE 3:** Correlations between different variables and the ISI score in our IBS population.

Variables (*n* = 700)	Pearson *r* correlation with ISI score (*p*)
Age	0.08 **(0.038)**
Sex	−0.01 (0.894)
BMI	0.04 (0.347)
Number of cigarettes per day	0.10 **(0.008)**
Recreational substances	0.05 (0.165)
Number of glasses per week	0.01 (0.753)
FD	0.22 **(< 0.001)**
GERD	0.10 **(0.008)**
Fibromyalgia	0.15 **(< 0.001)**
Endometriosis	0.08 **(0.037)**
IBS‐SSS	0.24 **(< 0.001)**
PHQ‐15	0.52 **(< 0.001)**
Pagi‐Sym (*n* = 660)	0.35 **(< 0.001)**
HAD‐A	0.42 **(< 0.001)**
HAD‐D	0.41 **(< 0.001)**

*Note:* Results are presented as Pearson *r* coefficient (*p*), bold values = significant. The number of cigarettes and alcoholic drinks consumed was calculated only for people who smoke and/or consume alcohol.

Abbreviations: BMI, Body Mass Index; FD, Functional Dyspepsia; GERD, gastroesophageal reflux disease; HAD‐S, Hospital anxiety (HAD‐A) and depression (HAD‐D) scale; IBS‐SSS, IBS Severity Scoring System; PAGI‐SYM: Patient Assessment of Upper Gastrointestinal Disorders‐Symptom Severity Index; PHQ‐15, Patient Health Questionnaire‐15.

A hierarchical linear regression was conducted to examine the relative contribution of the independent variables (that were significantly correlated with ISI in Table 3) to the prediction of the dependent variable (ISI score). In Table 4, Model 1 included control variables (age, number of cigarettes per day), Model 2 included gastrointestinal comorbidities (FD, GERD), Model 3 included severity of IBS (IBS‐SSS), and Model 4 included extra‐intestinal comorbidities (endometriosis, fibromyalgia, anxiety, depressive symptoms). PHQ‐15 and PAGI‐SYM were not included in the regression model to avoid overlap with other variables. PAGI‐SYM assesses gastrointestinal symptoms that overlap with FD and GERD, while PHQ‐15 reflects overall somatic symptom burden, which overlaps with symptoms of IBS (related to abdominal pain and bowel transit) and comorbid conditions such as fibromyalgia and sleep disturbances.

**TABLE 4:** Hierarchical linear regression with ISI score as dependent variable (*n* = 700).

	*B* (95% CI)			
	Model 1	Model 2	Model 3	Model 4
Age	0.036* (0.005–0.067)	0.036* (0.005–0.066)	0.037* (0.007–0.066)	**0.030** ^*^ (0.003–0.057)
Number of cigarettes per day	0.149** (0.005–0.067)	0.132* (0.031–0.233)	0.122* (0.023–0.221)	0.052 (−0.039–0.143)
FD		2.604*** (1.660–3.549)	1.988*** (1.034–2.942)	**1.693** ^***^ (0.828–3.558)
GERD		0.894 (−0.169–1.958)	0.784 (−0.260–1.828)	0.166 (−0.783–1.114)
IBS‐SSS			0.012*** (0.007–0.016)	**0.004** ^*^ (0.000–0.008)
Fibromyalgia				**1.880** ^**^ (0.634–3.126)
Endometriosis				0.982 (−0.167–2.130)
HAD‐A (Anxiety)				**0.389** ^***^ (0.282–0.496)
HAD‐D (Depression)				**0.315** ^***^ (0.197–0.433)
Adjusted R^2^	0.015	0.061	0.096	0.265
F	6.219**	12.395***	15.879***	28.980***

*Note:* Adjusted R^2^ corrects for the number of predictors in the model and the sample size.

Abbreviations: F Change, F test of variance gain; FD, Functional Dyspepsia; GERD, gastroesophageal reflux disease; HAD‐A and D, Hospital Anxiety and Depression Scale; IBS‐SSS, IBS Severity Scoring System; R^2^, total variance explained by the current model.

* *p* < 0.05.

** *p* < 0.01.

*** *p* < 0.001.

Model 1 explained only 1.5% (Adjusted R^2^ = 0.015) of the variance in the ISI score in IBS patients. Adding gastrointestinal comorbidities (FD and GERD) to model 2 significantly increased the explained variance (Adjusted R^2^ = 0.061, *p* < 0.001). Model 3 showed a significant impact of IBS severity in relation to the ISI score (B = 0.012, *p* < 0.001). Finally, the addition of extra‐intestinal comorbidities improved the explanation of the variance of the model to 26.5% (Adjusted R^2^ = 0.265, *p* < 0.001), which suggests an independent association between insomnia severity and older age, higher IBS severity, the presence of fibromyalgia and FD, and higher scores of anxiety and depressive symptoms.

## 4 Discussion

In our study, the prevalence of insomnia was found in more than 1/3 (38.6%) of our IBS population. This subgroup presented more severe gastrointestinal symptoms, lower quality of life, and higher burden of somatic and psychological comorbidities. Age, FD, IBS severity, fibromyalgia, and anxiety and depressive symptoms were independently associated with insomnia severity.

This prevalence aligns with previous findings reporting that about 37.6% of IBS patients experience sleep disorders, including insomnia, parasomnia, and hypersomnia [8], our study focusing exclusively on insomnia. This apparent similarity may be explained by the characteristics of our sample, drawn from a tertiary care center where patients often present with more severe IBS symptoms (75% had moderate to severe digestive symptoms according to the IBS‐SSS). Greater symptom severity is associated with psychological distress and hyperarousal, both potentially linked to insomnia [18]. Thus, the elevated prevalence probably reflects the overall disease burden rather than a narrower definition of sleep disorder.

Traditionally, IBS patients do not present gastrointestinal symptoms at night, and the presence of nocturnal symptoms should lead to the elimination of an organic condition via complementary examinations [19]. Based on the ISI score alone, external or environmental causes cannot be ruled out, but we found comorbidities to be significantly more common (*p* < 0.001) among patients with IBS affected by insomnia, confirming previous findings [2]. Among these, FD and its subtypes (EPS and PDS) affected around 60% of our population, consistent with rates observed in tertiary care and prior studies using Rome III criteria (64%) [20]. Similar associations between insomnia and FD (rather than IBS alone) have been noted; it was observed in another study that 61% of FD patients were affected by insomnia (with higher severity of anxiety and depressive symptoms) [21]. In our cohort, FD, fibromyalgia, and endometriosis were associated with the presence of insomnia, as reported previously [22, 23].

Univariate analyses showed that patients with IBS and insomnia were more likely to be smokers and to report higher levels of somatic symptom severity compared to those without insomnia. A meta‐analysis published in 2021 reported that regular smoking was significantly associated with an increased risk of insomnia [24], an effect likely mediated by nicotine, which enhances alertness and maintains physiological arousal. Regarding somatization, previous studies have consistently reported an association between insomnia and increased somatization [25, 26, 27, 28]. This relationship may be explained by shared underlying mechanisms, as sleep disturbances can alter pain modulation [29] and increase hypervigilance [30] to bodily sensations, thereby amplifying the perception and reporting of somatic symptoms.

Hierarchical linear regression analysis showed that psychological distress and somatic comorbidities (notably fibromyalgia, anxiety, and depressive symptoms) substantially improved the explanatory power of the model for insomnia severity. This suggests that insomnia in IBS might be less driven by gastrointestinal symptom severity than by the cumulative burden of somatic symptoms and psychological factors.

These findings support the growing evidence for a gut‐brain‐sleep axis, involving central sensitization, dysregulation of the HPA axis and autonomic nervous system imbalance [31], mechanisms likely intensifying both visceral sensitivity and hyperarousal, via a correlation between the severity of IBS symptoms, psychological distress, and insomnia, which indirectly support the physiological mechanisms described. Sleep disturbances may play a role in the exacerbation of IBS symptoms through central mechanisms, including increased visceral hypersensitivity, altered pain modulation, and dysregulation of the HPA axis [32, 33]. Conversely, IBS and its comorbidities, particularly those related to psychosocial impairment, may also contribute to sleep disturbances, notably through stress‐related mechanisms and potential alterations in gut microbiota [34]. This supports a bidirectional relationship, consistent with the gut‐brain interaction framework, in which sleep disturbances and gastrointestinal and psychological symptoms mutually reinforce each other. However, longitudinal studies are needed to clarify the temporal sequence and causal pathways underlying these associations.

Nevertheless, our study has several limitations, notably, the absence of a control group without IBS. However, a population‐based study conducted in a Korean sample representative of the Korean general population reported a 10.8% prevalence of insomnia using the ISI with a ≥ 10 cutoff. Although our study was conducted in a French sample, the prevalence of insomnia we observed was higher despite the use of a more stringent cutoff (≥ 15), which strengthens the robustness of this comparison. This suggests that even under stricter criteria, patients with IBS exhibit a higher prevalence of insomnia compared to the general population. This is also consistent with previous meta‐analytic findings suggesting an increased prevalence of sleep disturbances in patients with IBS compared to healthy controls [8]. Another limitation is the recruitment from a single tertiary center, which may not be representative of the global IBS population or patients seen in primary care. Furthermore, as our study relies on validated self‐report instruments (reflecting patients' perceptions and experiences), future research incorporating objective sleep measures (e.g., polysomnography) is warranted, as subjective and objective measures may diverge [35].

Despite these limitations, our study also has many strong points: firstly, we have a large sample of patients with IBS (*n* = 700), which ensures more reliable results. Secondly, we only used validated questionnaires, and comprehensive assessment of comorbidities strengthens the robustness of our findings.

Following our findings, gastroenterologists should be encouraged to systematically assess sleep quality as part of IBS management. Psychological and behavioral interventions, such as cognitive‐behavioral therapy for insomnia (CBT‐I) have shown promise improving both sleep and gastrointestinal symptoms, supporting an integrated therapeutic approach. This approach was validated via a randomized controlled trial with young students with IBS in Korea [36], which targets hypervigilance as well as emotional dysregulation linked to sleep problems, which are highly relevant in IBS. Additionally, gut‐directed hypnotherapy has been shown to be effective in the management of IBS, improving the patients' overall symptoms [37], psychological well‐being [38], and sleep [39]. Combined with treatments for diarrhea/constipation and antidepressants, this could greatly improve their quality of life and their IBS symptoms.

In conclusion, insomnia in IBS is a multifactorial condition, primarily linked to psychological distress and somatic comorbidities, such as anxiety and depressive symptoms, FD and fibromyalgia rather than gastrointestinal symptoms alone. Patients with IBS and insomnia exhibit more severe overall symptoms and poorer quality of life. Whether this results from a subjective deterioration in health perception or from the higher comorbidity load remains uncertain. These results underscore the importance of integrating sleep assessment and management into the routine care of IBS. Addressing insomnia through psychological and behavioral interventions may improve both mental and gastrointestinal outcomes.

## Author Contributions

C.M. and C.D. planned the study, H.M.‐M., M.N., A.‐M.L., C.M. and G.G. conducted the study, M.N., G.G., A.‐M.L., C.D. and C.M. collected data, E.A., H.M.‐M., I.A.T., F.B. and C.M. interpreted the data, and all authors drafted the manuscript. All authors have approved the final draft submitted.

## Funding

The authors have nothing to report.

## Ethics Statement

To be included, all participants had to be 18 years of age or older, have given a written informed consent. All patients are part of the cohort from the research protocol OTFI “OBSERVATOIRE DES PATIENTS SOUFFRANT DE TROUBLES FONCTIONNELS INTESTINAUX”, approved by Ile‐de‐France VII Committee for the Protection of Persons board in 2019 (N° ID‐RCB: 2017‐A02134‐49).

## Conflicts of Interest

C.M. has served as a consultant/advisory board member for KyowaKirin, Norgine, Biocodex, Mayoly Spindler, Tillots, Ipsen, Nestlé HealthScience, Reckitt, Viatris, and De Opella. A.‐M.L. served as a consultant for Medtronic. F.W. received consulting and/or lecturing fees from Biocodex Belfium, Biocodex France, Grünenthal, Menarini Belgium, Sanofi, and Viatris.

## Data Availability

The data that support the findings of this study are available from the corresponding author upon reasonable request.
